# Raman Scattering-Based Biosensing: New Prospects and Opportunities

**DOI:** 10.3390/bios11120512

**Published:** 2021-12-13

**Authors:** Kseniya V. Serebrennikova, Anna N. Berlina, Dmitriy V. Sotnikov, Anatoly V. Zherdev, Boris B. Dzantiev

**Affiliations:** A.N. Bach Institute of Biochemistry, Research Center of Biotechnology, Russian Academy of Sciences, 119071 Moscow, Russia; ksenijasereb@mail.ru (K.V.S.); berlina.anna@inbi.ras.ru (A.N.B.); sotnikov-d-i@mail.ru (D.V.S.); zherdev@inbi.ras.ru (A.V.Z.)

**Keywords:** Raman spectroscopy, coherent anti-Stokes Raman spectroscopy (CARS), stimulated Raman spectroscopy (SRS), resonance Raman spectroscopy (RRS), surface-enhanced Raman spectroscopy (SERS), nanoparticles, optical sensors, immunosensors, signal enhancement, lateral flow test strips

## Abstract

The growing interest in the development of new platforms for the application of Raman spectroscopy techniques in biosensor technologies is driven by the potential of these techniques in identifying chemical compounds, as well as structural and functional features of biomolecules. The effect of Raman scattering is a result of inelastic light scattering processes, which lead to the emission of scattered light with a different frequency associated with molecular vibrations of the identified molecule. Spontaneous Raman scattering is usually weak, resulting in complexities with the separation of weak inelastically scattered light and intense Rayleigh scattering. These limitations have led to the development of various techniques for enhancing Raman scattering, including resonance Raman spectroscopy (RRS) and nonlinear Raman spectroscopy (coherent anti-Stokes Raman spectroscopy and stimulated Raman spectroscopy). Furthermore, the discovery of the phenomenon of enhanced Raman scattering near metallic nanostructures gave impetus to the development of the surface-enhanced Raman spectroscopy (SERS) as well as its combination with resonance Raman spectroscopy and nonlinear Raman spectroscopic techniques. The combination of nonlinear and resonant optical effects with metal substrates or nanoparticles can be used to increase speed, spatial resolution, and signal amplification in Raman spectroscopy, making these techniques promising for the analysis and characterization of biological samples. This review provides the main provisions of the listed Raman techniques and the advantages and limitations present when applied to life sciences research. The recent advances in SERS and SERS-combined techniques are summarized, such as SERRS, SE-CARS, and SE-SRS for bioimaging and the biosensing of molecules, which form the basis for potential future applications of these techniques in biosensor technology. In addition, an overview is given of the main tools for success in the development of biosensors based on Raman spectroscopy techniques, which can be achieved by choosing one or a combination of the following approaches: (i) fabrication of a reproducible SERS substrate, (ii) synthesis of the SERS nanotag, and (iii) implementation of new platforms for on-site testing.

## 1. Introduction

**Figure 1:**
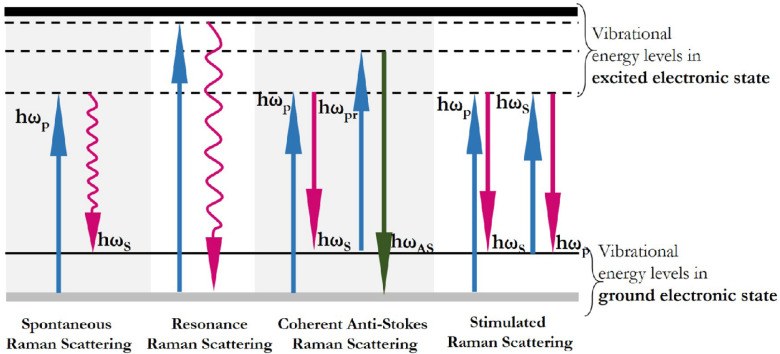
Energy level diagram demonstrating the Raman, RRS, CARS, and SRS processes.

Currently, Raman spectroscopy is a promising analytical tool that provides a chemical fingerprint for molecular identification [1,2]. Raman spectroscopy relies on inelastically scattered light and allows for the identification of vibrational states (phonons) of molecules. The phenomenon of inelastic light scattering by molecules was observed for the first time in 1928 by the group of the Indian scientist Raman [3]. Most of the scattered light does not change in frequency when photons of light interact with a substance (Rayleigh scattering). However, under incident light, inelastic light scattering processes can also occur, resulting in the emission of scattered light with more or less frequency (anti-Stokes and Stokes bands, respectively) due to molecular vibrations [4]. Figure 1 shows a diagram of energy levels and transitions corresponding to the processes of inelastic and Rayleigh light scattering. Thus, a Raman spectrum is formed, consisting of bands, the position of which depends on the vibrational frequencies that are characteristic of each functional group of the sample molecules. The widespread use of Raman spectroscopy and its integration into a number of analytical methods occurred much later than the discovery of the effect of inelastic scattering, only in the 1960s, with the advent of commercially available lasers to excite the sample [5,6]. Currently, Raman spectroscopy is successfully applied for the qualitative and quantitative determination of unknown compounds in complex samples [7,8], as well as for the registration of structural changes [9,10].

Despite its speed, accuracy, and reliability, the weak point of spontaneous Raman spectroscopy is the rather low scattering cross-section of ordinary molecules, resulting in a weak signal. Moreover, the application of Raman spectroscopy requires individual optimization of research parameters, including excitation lasers, a filtering mechanism, and an objective lens, which depend on the object of study. The above factors have boosted the development of Raman techniques, of which there are now more than 25 types [11], including Raman techniques based on resonant [12,13], coherent [14,15], surface-enhanced [16,17,18], and tip-enhanced [19,20] Raman scattering phenomena.

The discovery of different types of Raman techniques provided an enormous stimulus to biomedical scientific and applied research because the spectrum of scattered photons for each molecule is unique, allowing for easy identification of a matter of interest. Moreover, Raman spectroscopy provides a number of advantages, such as noninvasiveness, no need for sample preparation, the ability to work with aqueous samples, and the possibility of combining these with other methods of analysis. The nondestructiveness of the method makes it suitable for in vivo analysis and diagnosis, providing information about the structure, conformation, and interaction of biomolecules [21]. Thus, the effectiveness of Raman spectroscopy in establishing the composition and functions of the components of the photosystem was shown, which provides an understanding of the detailed mechanisms of photosynthesis [22,23]. Beyond this, Raman techniques are a promising tool for creating chemically selective hyperspectral images, allowing thousands of Raman spectra to be obtained from the whole field of view, for example, by scanning a focused laser beam through a sample. The resulting data can be used to image the location and number of different components. By analyzing biological samples, hyperspectral imaging can show the distribution of proteins, lipids, nucleic acids, and carbohydrates [21,24,25,26]. Previously, there have been reviews of recent developments in Raman spectroscopy in relation to various objects of research [27,28,29] and areas of method applications [30,31,32,33].

In this review, we focus on Raman spectroscopy techniques that are most commonly used in life science research, including coherent anti-Stokes Raman spectroscopy (CARS), stimulated Raman spectroscopy (SRS), resonance Raman spectroscopy (RRS), and surface-enhanced Raman spectroscopy (SERS), to assess the detection capabilities of and prospects for applying these Raman techniques in the biosensing field. In Section 1, we briefly discuss the fundamental principles of the selected techniques and overview their application for analysis of biological samples regarding the advantages they offer over all Raman spectroscopy techniques. RRS, one of the Raman enhancement techniques, allows for studying the structural elements of chromophores, amino acids, and proteins by adjusting the excitation wavelength in the absorption band of individual elements. It has enormous potential for studying biomolecules [34,35]. Other Raman enhancement techniques, namely, nonlinear Raman spectroscopy, which is based on coherent anti-Stokes Raman scattering or stimulated Raman scattering, provide a higher signal sensitivity in the spectra with a cutoff of the possible fluorescence of the sample, which also makes these techniques promising for studies of biomolecules [36,37]. The limitations of nonlinear Raman techniques and RRS, as well as possible ways to overcome them to achieve the required characteristics, are considered in parallel. However, despite the improvement of the signal up to eight orders of magnitude achieved using the above techniques, the largest signal enhancement up to 14 orders of magnitude is provided by SERS. Consequently, the future development of these techniques for bioanalytical applications lies in their combination with SERS. 

In Section 2, we focus on the fabrication of SERS-active substrates and their recent application in surface-enhanced nonlinear and resonance Raman techniques for the analysis of biological samples with an assessment of their potential for immunoanalytical applications. However, it should be noted that the use of surface-enhanced coherent Raman techniques is limited to molecular imaging of functional molecules in tissues and cells. Therefore, among the existing variety of enhancement Raman techniques, SERS remains the most studied and widely used analytical tool for biosensor purposes. In this regard, most of Section 2 is devoted to approaches to the fabrication of SERS substrates and SERS nanotags, as well as the implementation of various receptor molecules for the development of biosensor systems. 

Despite the fact that SERS is a well-studied and widespread method for creating biosensors, its adaptation for analysis of real samples and on-site monitoring is limited by insufficient signal reproducibility and accuracy. These limitations can be removed by integrating SERS into a microfluidic platform, as well as combining the high sensitivity of SERS with the advantages of other methods to create a high-performance biosensor system. Recent advances in the development of SERS chips and a combination of multiple methods for high-throughput analysis are discussed in Section 3.

Miniaturization and simplification of readout devices are a major thread in successful implementation of Raman spectroscopy in real-life practice. Of particular interest are portable Raman spectrometers for on-site sample identification. In Section 4, we discuss the successful development of portable devices for implementing the Raman spectroscopy techniques discussed in this review.

### 1.1. Resonance Raman Spectroscopy

One of the approaches to amplify the Raman signal is to use the resonant excitation frequency. Resonance Raman scattering is observed when the frequency of the incident light coincides with one of the frequencies of the electronic transition of the molecule under study (see second combination of energy levels in Figure 1). The given accordance of frequencies leads to significant enhancement of optical signals as compared with common Raman scattering (for which only a very small fraction of the exciting photons causes radiation). In the case of RRS, the features of electronic absorption determine the intensities of various Raman lines and thus change the ratio of these intensities. By this way, the RRS spectra, in contrast to the common Raman spectra, depends on the exciting line [38]. Consequently, the excitation wavelength in the case of RRS varies for individual molecules. The characteristic features of this technique include: (i) a high gain, which is 3–8 orders of magnitude higher than the intensity of spontaneous Raman scattering; (ii) selective amplification of some normal vibrations relevant for a given electronic absorption band; (iii) in some instances, the effect of the progressions of intense overtones in resonance Raman (RR) spectra, and the connection between RR line intensity and the parameters of the electronically excited state [39,40]. Taken together, these advantages allow RRS to be considered an effective technique for studying the vibrational and electronic structure of chromophores within a biomolecule or the entire cell and to be used in the field of life sciences and medicine to study a particular chromophore that plays a key role in bioprocesses (e.g., to study cellular changes during bioprocesses). Thus, RRS was first applied to study the changes during the aggregation of a light-harvesting complex of photosystem II under illumination [40]. Since then, research in the field of photosynthetic processes has continued, with a number of works describing the mechanisms of photoprotection and photoinactivation provided by chlorophyll and carotenoid molecules [23,41,42]. The RRS showed high selectivity to the chromophore centers of heme proteins because excitation in the UV (UVRRS) and visible regions allows one to enhance the vibrational bands of the chromophore element and to study aromatic protein residues and peptide bonds, respectively, by tuning the laser to the wavelength of the electronic transition [43,44,45].

Another practical area in which the RRS technique has found application is the monitoring of antibody titers during the production of recombinant antibodies in cell cultures [46]. In this case, RR spectra provide information on changes in the composition of proteins and nucleic acids to characterize growth as well as the quality and quantity of the product. Despite the aforementioned sensitivity, specificity, and minimization of the effect of fluorescence, it is worth noting the drawbacks of this technique. The process of photodegradation inside the sample due to excitation under resonant Raman conditions requires the rapid movement of the sample under the illuminating beam, which does not allow combining RRS with microscopy for visualizing biological samples [47]. Another challenge consists of the need for expensive and complex equipment, including spectrometers, Rayleigh rejection filters, and powerful lasers, which hinders the practical implementation of the method.

### 1.2. Coherent Raman Spectroscopy

The application of incident lasers in spectroscopic methods allows for the generation of such an electromagnetic field density in the sample that the response of the light substance becomes nonlinear [48,49]. In this case, the displacement of the charged particle from the equilibrium position and, accordingly, the polarization of the medium, which is a secondary source of radiation, occurs not in direct proportion to the applied field, but with a deviation from the linear dependence. Under these conditions, the generation of various optical harmonics arises, including coherent anti-Stokes and stimulated Raman scattering, which are the subject of nonlinear optics. CARS and SRS are the most common techniques and are considered promising tools for studying molecular distributions in cells and tissues, as well as for visualizing biological structures [50,51]. CARS is a third-order nonlinear optical process in which photons of pump frequency ω_p_, the Stokes frequency ω_S_, and probe frequency ω_pr_ interact in such a way that a signal is generated in the anti-Stokes field at a frequency of ω_AS_ = (ω_p_ − ω_S_) + ω_pr_ when the frequency difference ω_p_ − ω_S_ matches the Raman-active molecular vibration Ω_R_. In other words, the system uses a sequence of laser sources. First, laser radiation, as in conventional Raman spectroscopy, transforms the molecule into an excited state. The second scanning laser detects such excited molecules and shifts the final radiation. As a result, the final signal is shifted in the blue region of the spectrum. Compared to spontaneous Raman scattering, the CARS signal is several orders of magnitude higher and is shifted to the blue field relative to the excitation waves, which provides spectra free from the background fluorescence of the sample [15,52]. Duncan et al. [53] were the first who designed the CARS microscope to study onion skin cells. However, the initial steps toward the application of this method as a label-free imaging technique of biomolecules were only made at the end of the 1990s with the invention of modern CARS microscopes [54]. Further development of the CARS technique was aimed at suppressing the nonresonant background affecting CARS measurements. Therefore, to reduce the nonresonant contribution to the signal, researchers have proposed several approaches, including the use of time-resolved CARS [55,56], spectral interferometric polarization CARS [57], epi-detection of CARS signal [58], multiplex CARS [59,60], and deep learning models to extract vibrational information from spectra [61].

**Figure 2:**
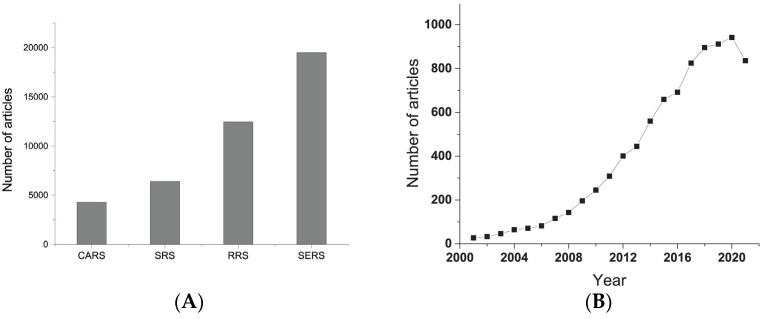
(**A**) The number of articles published over the past two decades (January 2001–October 2021) according to the Web of Science (WoS) database found using the following keywords: (a) coherent Raman scattering; (b) stimulated Raman scattering; (c) resonance Raman scattering; (d) surface-enhanced Raman scattering. (**B**) Dynamics of published articles on the SERS over the past two decades (January 2001–October 2021) according to the WoS database.

Over the past two decades, not many studies have been published covering nonlinear Raman techniques (Figure 2A), but there is undoubtedly interest in this area of research, which is evidenced by the successful application of CARS for the label-free visualization of lipid, protein, and nucleic acid distribution in cells [60,62]; pigments in living cells [63]; and skin tissues [64]. The CARS technique is of particular importance in the field of cancer cell identification and diagnosis research [65,66,67].

The SRS microscopy is similar to CARS and was first implemented using two low-power continuous-wave lasers to acquire Raman spectra in liquid benzene [68]. Much later, in 2008, the capabilities of SRS-based microscopy for biomedical purposes were demonstrated [69]. SRS is based on the interaction of two beams, a Stokes (ω_S_) and a pump (ω_p_), resulting in the stimulated excitation of the vibrational transition when the frequency difference coincides with the Raman active frequency of the sample (Figure 1). The advantages of SRS over CARS include the absence of a nonresonant background, and the position of the spectral lines completely coincides with those in the spontaneous Raman spectrum. Moreover, the intensities of the SRS signal are directly proportional to the concentration of the substance, which makes it a perspective method for quantitative detection. The use of pump laser with a constant frequency and a scanning Stokes laser provides unique responses of molecules after double excitation, which is the difference between SRS and other types of Raman scattering. When the pump energy matches the difference between excited and ground energy states of the molecule, it causes an increase in the generated signal by orders. The effectiveness of SRS microscopy for image interpretation and the quantification of complex biologic specimens has been confirmed in a number of recent studies [70,71,72].

For studies of SRS and CARS microscopy, ultrafast laser-pulsed systems are required to excite the sample, but the high cost of such systems prevents their widespread use. In addition, the obtained spectra of various compounds will qualitatively differ from the Raman spectra, which narrows their field of application to visualization and does not allow these techniques to be widely used in current biosensing technologies.

### 1.3. Surface-Enhanced Raman Spectroscopy

Compared to the techniques described above, SERS is a continuously evolving field of research and is of particular interest for the identification of trace molecules. The SERS phenomenon, which arises as a result of signal amplification on the substrate surface due to the excitation of plasmon resonances, was first discovered in 1974 by Fleischmann and his group [73]. However, the interpretation of the observed phenomenon was obtained only in 1977 as a result of studies on the spectra of pyridine, nitrogen heterocycles, and amines on the silver surface [74]. Following these publications, a study was conducted on the SERS detection of single molecules [7,8], which prompted the study of the enhancement mechanism of Raman scattering. To date, two mechanisms have been proposed, electromagnetic and chemical enhancement, which determine a huge increase in the Raman scattering cross section of adsorbed molecules. It was stated that the electromagnetic mechanism makes the greatest contribution and provides in some cases up to a 10^10^ amplification of Raman scattering [75]. The electromagnetic mechanism is associated with an increase in the electromagnetic field upon the excitation of localized surface plasmons. In this case, the resonant frequency of conduction electrons in a metallic nanostructure depends on the size, shape, and roughness of the nanomaterial. Examples of reproducible substrate surfaces for SERS that provide significant amplification of the electromagnetic field will be discussed below. The enhancement factor provided by the chemical mechanism is approximately 10^3^ and stems from changes in the electronic structures of molecules near the metal surface during chemical interaction [76,77].

Understanding the nature of the Raman enhancement effect gave impetus to the development of SERS and the combination of SERS with coherent nonlinear Raman techniques and RRS, as well as their application in life sciences. Scientific research in the field of SERS demonstrates the growing interest in the technique associated with its high sensitivity, the ability to detect molecules by characteristic spectra, simplicity of sample preparation, and rapidity due to the unique ability to amplify the Raman signal up to 15 orders of magnitude [78,79]. The intensive development of the method over the past 20 years (see Figure 2B) has been facilitated by advances in the synthesis and modification of nanoscale SERS-active materials and the improvement of devices, including the advent of handheld spectrometers. Thus, the development of technologies has made it possible to observe the Raman scattering of small volumes of molecules up to single-molecule detection [9], and to increase the resolution, which both open the possibility for in vivo and in vitro biosensing. Several reviews have demonstrated the potential of SERS for the detection of oligonucleotides, proteins, and tumor cells in biofluids [10,30,31].

As a result of the assumption of the possibility of achieving single-molecule detection by Raman techniques, the combination of enhanced local fields near metal surfaces with the resonant effect of Raman scattering or coherent Raman scattering allowed for the development of the following techniques: SERRS, SE-SRS, and SE-CARS. The contribution of plasmonic effects of metal nanostructures in the form of films or colloidal nanoparticles was estimated by studying the enhancement of the coherent and resonance Raman signal for various biological objects [34,36,37,43,78,80].

Despite the advances of SERS, the practical implementation of the SERS-combined techniques requires standardized procedures for obtaining a uniform substrate surface and detecting a signal from a molecule of interest in a hot spot area, which remain urgent tasks today. Moreover, the analysis of complex samples containing a variety of biomolecules complicates the identification of the target molecule. In this regard, the performance of Raman techniques can be greatly amplified through the use of bioreceptor molecules with specificity for the target molecule. One such example is a SERS-based biosensing platform that combines the specificity of antigen–antibody interactions with the sensitivity of SERS. Below, we consider the current developments for the high-performance detection and characterization of biological objects, including the variety of metal substrates and SERS nanotags as well as SERS-based biosensor applications.

## 2. Application of SERS and SERS-Combined Raman Techniques in the Detection of Biomolecules

**Figure 3:**
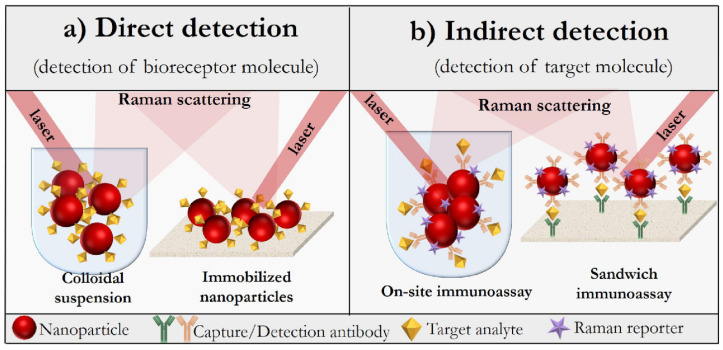
Schematic representation of the direct (**a**) and indirect (**b**) approaches for SERS-based detection.

Generally, SERS-based detection is divided into two approaches, that is, direct or label-free detection and indirect detection, using Raman reporter molecule-labeled SERS nanotags [76,79]. The label-free approach allows for the obtaining of the Raman spectra of biomolecules adsorbed on nanostructured SERS-active substrates. The principle of this approach is schematically illustrated in Figure 3a of this review. In order for the specificity of detection to be improved, various bioreceptor molecules, including antibodies and aptamers, which specifically bind the analyte, are immobilized on a metallic nanostructured substrate. In this case, the sample is identified by comparing the Raman spectra before and after the binding of the target molecule. However, in the direct approach, any changes in the biomolecule’s environment contribute to the Raman spectrum, which complicates the use of direct detection in complex sample matrices due to the influence of the background signal. Moreover, not all types of analytes can be discriminated against their own spectra of molecules [81]. 

On the contrary, in the indirect approach, specific target detection is performed by detecting the vibration spectrum of a Raman reporter molecule, which is a chemical compound, most often a dye or sulfo-derivatives of the aromatic series, with a rather large scattering cross-section, resulting in intensive bands of the SERS spectrum. This approach is illustrated in Figure 3b. The high sensitivity is achieved due to the choice of a suitable reporter molecule and its proximity to the SERS nanotag. However, the indirect approach also has its disadvantage associated with the impossibility of obtaining molecular information about the target biomolecule itself. To summarize, depending on the objectives pursued and the sample matrix, a particular Raman technique should be chosen from the existing variety of methods, followed by the design of a suitable plasmonic nanostructured substrate or SERS nanotag [82]. This chapter aims to discuss the assembling of direct and indirect SERS-based platforms for the characterization and detection of various molecules with the potential for their use in the biosensor field.

### 2.1. Direct and Indirect Approaches of SERS-Based Techniques

In the direct approach of SERS, the sensitivity and specificity of the system are determined by the properties of the substrate and the functionalization of its surface, respectively. As stated earlier, the phenomenon of SERS is based on the enhancement of the local field provided by the surface plasmon resonances of metallic nanostructures. The enhancement effect appears when a biomolecule is adsorbed or is located near the surface of a metal substrate (the distance should be less 30 nm) [83].

**Figure 4:**
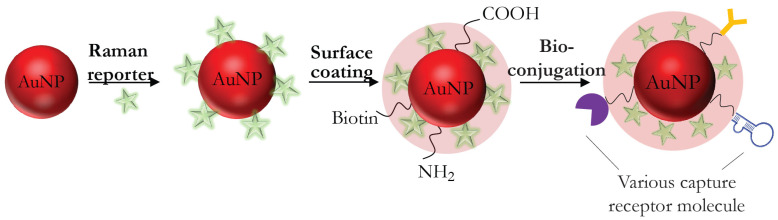
Design of SERS nanotag consisting of a metal nanoparticle core, modified by reporter molecule (green stars), protected by biocompatible layer (pink shell), and functionalized by a capture receptor molecule.

In most cases, the SERS-based technique application involves an indirect approach for the identification and quantitative detection of molecules because it allows the achieving of the necessary sensitivity for the determination of analytes with a small Raman scattering cross section. A distinctive feature of the indirect approach is the use of the SERS nanotag, which provides the formation of the SERS signal when it binds to the target analyte. The SERS nanotag contains the following components: (i) a metallic nanostructured substrate, (ii) a protective shell or layer that ensures the stability and biocompatibility of the SERS nanotag (the nonessential component of the SERS nanotag), (iii) capture receptor molecule, and (iv) the Raman reporter molecule responsible for the formation of the fingerprint spectrum. Figure 4 illustrates the structure of the SERS nanotag typically applied to design the indirect SERS detection. The indirect SERS techniques offer unique opportunities for qualitative and quantitative analysis due to the variety of SERS nanotags and sensor design. In order for the insufficient stability of colloidal nanoparticles and the irreproducibility of the Raman signal associated with the desorption of reporter molecules from the surface of nanoparticles or competition for binding with other molecules to be eliminated, a protective shell or layer is used in some cases.

Modification of the SERS nanotag with bioreceptor molecules that specifically bind the analyte gave rise to the development of SERS-based biosensors. To date, many successful studies have been published on the use of SERS nanotags in bioimaging and biosensors [84,85,86]. However, to date, the only example of the indirect detection using the SERS nanotag in nonlinear Raman spectroscopy has been described. The potential of SE-CARS using a SERS nanotag has been demonstrated for the visualization of the basal cell marker p63 location in prostate tissue sections [35]. In this work, the SERS nanotag included Au/Ag nanoshells modified with a reporter molecule 5,5′-dithiobis (2-nitrobenzoic acid) and stabilized with a protective layer of triethylene glycol with a terminal carboxyl group, through which bioconjugation with p63 antibodies was carried out. The visualization of the sample by CARS microscopy in combination with the SERS nanotag made it possible to localize tumor-relevant antigens in tissue samples selectively, sensitively, and quickly. 

In order for an indirect SERS approach based on antigen–antibody (or another receptor molecule) interactions to be implemented, a sandwich scheme for fabricating a SERS biosensor is traditionally applied. The sandwich scheme includes the following components: (i) the capture antibody (fixed on the substrate surface or freely located in solution), (ii) an antigen (the target analyte—it can be a protein, cell, etc.), and (iii) a labeled detection antibody. As noted above, at the moment, there is only one example of the development of immuno–SE-CARS, and the adaptation of SERS-combined nonlinear Raman spectroscopy techniques for indirect analysis of biomolecules is under development; therefore, further in the review, biosensors based on the indirect SERS approach will be considered.

To summarize, the direct approach allows for on-site identification and quantification of the analyte based on unique SERS fingerprint spectra. The direct approach is most preferable for on-site detection because the additional stage of obtaining the SERS nanotag, where the reporter molecule must be in close proximity to the surface of the noble metal nanoparticle, complicates the detection procedure. In addition, of particular interest is the ability to identify components in a mixture without a separation step. However, it should be noted that compounds of matrixes may interfere the registered spectra as well as at values of specific spectral peaks. In this case, preference is given to the indirect approach, where the analyte is captured using a receptor molecule and the extrinsic SERS signal is acquired from the reporter molecule, which together reduces the contribution of interfering components to the determination of the target analyte. Therefore, the choice of a suitable approach for SERS detection is based on the purpose of the study, as well as the matrix of the sample in which the analyte is identified.

#### 2.1.1. Substrate Fabrication

Great efforts have been made to develop various types of nanostructured substrates, which are characterized by a high enhancement factor (EF) [48,49,87,88,89]. The most common materials for the formation of colloidal and solid substrates are silver and gold nanoparticles due to their unique optical and electronic properties, exhibiting plasmon resonance bands in a wide range of spectra from the visible to the near infrared. Varying the size and shape of nanostructures leads to a change in the amplitude, position, and width of the surface plasmon resonance (SPR) line. The variety of substrates described in the literature can be divided into two groups: colloidal substrates and solid substrates. The traditional methods of the synthesis of colloidal metal substrates include the chemical reduction of metal salts or laser ablation. Currently, methods of synthesis and the application of various nanostructures, including nanospheres [35], nanovoids [90], nanostars [91,92], and nanoflowers [93] in SERS-based techniques, are described. The signal enhancement efficiency is influenced by the size and shape of the metal nanoparticles. Thus, nanoparticles less than 100 nm in size are considered optimal for the fabrication of SERS substrate because when large nanoparticles are irradiated with a laser, multipole excitation arises, which is nonradiative and reduces enhancement efficiency. When choosing the shape of metal nanoparticles, preference is given to anisotropic structures, such as star-shaped nanoparticles, which, due to their unique optical properties, are widely used in biosensors and imaging techniques [94]. Of particular interest is the aggregation of colloidal metal nanoparticles, which provides a high EF. For example, the random aggregation of spherical and complex nanoparticles can lead to the formation of regions with a strongly enhanced electromagnetic field, known as “hot spots” [95]. When the biomolecule is close to this region, the Raman enhancement increases up to 10^15^ orders of magnitude, and a single-molecule detection can be achieved [96]. However, it should be noted that an aggregated colloidal substrate can lead to an unreproducible and unstable signal, which is critical for bioanalytical applications of SERS and SERS-combined techniques.

Compared to colloidal metal substrates, solid substrates are characterized by homogeneity; ease of sample preparation, including surface functionalization and washing steps; and the allowing of the detection of single molecules in a small volume. The formation of solid substrates is carried out by self-assembly [97,98,99] or lithography [100,101,102] methods or using the combination of these methods, resulting in the creation of substrates with different SPR properties. Table 1 summarizes various approaches to create SERS-active substrates [86,94]. The self-assembly is a cost-effective and rapid approach for creating a SERS-active solid substrate. The quality and efficiency of the SERS-active substrate in this case depends on the functional groups on the solid support, the incubation time of the support in the colloidal solution, the plasmonic properties of the nanoparticles, and their concentration. However, in a uniform distribution of nanoparticles with a controlled size and shape, providing an enhanced and reproducible Raman signal can be achieved using nanolithography. Since its inception, this technology has received a large amount of attention because it allows for the fabrication of uniform, periodic nanostructures of SERS substrates. Thus, silicon nanopillars modified with metal nanoparticles with a high surface-to-volume ratio have proven their effectiveness in detecting various biomolecules [103,104,105]. Today, special attention is also paid to the fabrication of SERS substrates using biomimetic natural materials as a template for the further distribution of plasmonic nanostructures. One of these natural templates is diatoms, which are unicellular solitary or colonial microscopic organisms with a shell over the cell membrane. The shell of diatoms is composed of amorphous silica, forming an ordered structure of hierarchical micro-nanopores. Cai and coauthors have shown a simple and cost-effective method for fabricating the SERS substrate based on the nanoimprinting technology to replicate the micro-nanopore pattern of the diatom shell on polydimethylsiloxane (PDMS) film [106]. After that, gold nanoparticles were sprayed onto the PDMS film with nanopillars, adjusting the size and gap between the particles by varying the duration of the process. The effectiveness of the SERS substrate was shown by the example of the detection of the pesticide carbendazim with high sensitivity.

Along with the advantages of noble metals, mention must be made of the laboriousness and high cost of the procedures for manufacturing (the lithography of nanospheres, electron beam lithography, etc.) SERS substrates based on them. In addition to noble metals, dielectrics and semiconductors are considered alternative materials for fabricating SERS substrates. In recent years, three-dimensional hybrid metal/semiconductor nanostructures have attracted much attention as reusable SERS substrates. When assembling a dielectric template or semiconductor structure with noble metal nanoparticles, the SPR frequency of the nanoparticles shifts to the IR region, resulting in an increase in SERS due to the coincidence of the biomolecule Raman signal with the SPR of nanoparticles. Pal et al. fabricated reusable Ag/ZnO/Au SERS substrates by using thermal evaporation, hydrothermal growth, and sputtering for the formation of silver film, ZnO nanorods, and gold nanoparticle deposition, respectively [107]. Under the optimized structure of a hybrid SERS substrate, the highly sensitive detection of λ-DNA was achieved. Among the promising semiconductor materials is titanium dioxide, which, after elemental doping with metallic and nonmetallic structures, forms a SERS-active substrate. Thus, Keshavarz et al. proposed a SERS substrate based on a quantum-structured (Q-structured) TiO_x_ template and TiO_2_ quantum particles with oxygen vacancies, which are self-assembled on the template [108]. Evaluation of the efficiency of the SERS substrate showed the nanomolar range of the epidermal growth factor receptor detection. In the work of Qu et al. [109], the etched glass substrates were used with the layer of immobilized gold nanoparticles, followed by a protective polydopamine coating and antibody immobilization. The implementation of multicomposites based on several types of nanoparticles in combination with a Raman reporter molecule made it possible to obtain a multilayer structure. The presence of nanosized cubes of Au–Ag on the surface of a silicon wafer made it possible to increase the number of “hot spots” and to improve the detection limit of alpha-fetoprotein in a sandwich immunoassay to levels [110].

Recently, organic semiconductor thin films have been found to be an alternative substrate for SERS because they increase SERS activity mediated by charge transfer between the substrate and the analyte [111]. Organic thin films have a number of advantages, including simple and fast synthesis, the ability to customize the optoelectronic properties, and the structural identity of the resulting films. Thus, Yilmaz et al. [112] confirmed the effectiveness of a substrate on the basis of organic semiconductor for SERS detection. They fabricated hydrophobic film based on α,ω-diperfluorohexylquaterthiophene (DFH-4T) by vapor deposition and applied it as a substrate for acquiring SERS spectrum of methylene blue, thereby achieving EF of 3.4 × 10^3^. The subsequent modification of the DFH-4T film with a thin gold layer resulted in an enhancement up to 10^10^ with the possibility of detecting a sub-zeptomole level of methylene blue. The achieved gain demonstrate that organic semiconductor films are promising for the fabrication of SERS substrates that allow the identification of low analyte concentrations down to a single molecule, avoiding expensive procedures for preparing substrates, and their application in various fields of research, including the development of biosensors. 

**Table 1:** Methods for fabricating SERS-active solid substrates, summarized according to the following reviews.

Method	Approach	Features	Ref.
Self-assembly (or bottom-up methods)	Chemical immobilization	Immobilization of the nanoparticles of various shapes and sizes through bifunctional reagents (linkers) containing amine or thiol groups on a solid support. The common bifunctional reagents are aminosilanes (APTES) and mercaptosilanes (MPTS).	[113,114]
	Electrostatic interaction	Electrostatic deposition of nanoparticles onto a polymer-modified solid support. The common polymers are poly (diallydimethylammonium chloride) (PDDA) and polyvinylpyrrolidone (PVP).	[98,115]
	Capillary force-induced assembly	The deposition of a colloidal solution of nanoparticles onto a solid surface, followed by the evaporation of the solvent-creating capillary force-induced assembly of metal nanoparticles.	[99,109]
	Langmuir film fabrication	Assembling of metal monolayer at an air–liquid interface, followed by the transfer of the film onto a solid support.	[97]
Lithography (or top-down methods)	Electron beam lithography	Design of metal pattern using an electron beam.	[100]
	Hole–mask colloidal lithography	Controlled self-assembly of colloidal nanoparticles serving as a mask for the formation of a metal plasmon pattern.	[101]
	Nanoimprint lithography	Fabrication of metal plasmonic structures on rigid and flexible substrates.	[106,116]

#### 2.1.2. Direct SERS-Based Biosensing

Pursuant to the definition given in [117] a label-free biosensor is a device that converts a cellular signal into a quantitative signal and was originally designed as an analytical tool for analysis of biomolecular interactions. The implementation of the direct SERS approach makes it possible to acquire the fingerprint spectrum of a substance under the condition of its immediate proximity to the active SERS substrate. Despite all the advantages of the direct detection approach, its prevalence is limited by the difficulty of obtaining reliable spectra of biomolecules, among other interfering components of biofluids. However, interest in measuring the intrinsic spectra of biomolecules is not diminishing because label-free detection provides information on the conformation and orientation of molecules and allows the analysis of molecular dynamic changes in bioprocesses and interactions between biomolecules and other compounds. To date, a successful application of the direct surface-enhanced Raman approach is confirmed in the detection of whole cells, bacteria, and proteins [80,118,119,120].

Recent developments of direct SERS-based biosensor systems are summarized in Table 2. To improve the reliability of the direct approach and enhance the binding of the analyte to the nanostructured substrate, researchers proposed a preliminary coupling reaction. Thus, in the study of Chen and coauthors [121], a direct SERRS is proposed, wherein the quantitative determination of the protein is carried through the formation of bicinchoninic acid (BCA)–Cu^+^ complex. The sensing mechanism included two stages: (1) the reduction of Cu^2+^ to Cu^+^ by proteins in alkaline conditions; (2) the formation of a chelate (BCA)–Cu^+^ complex. After a coupling reaction between the BCA–Cu^+^ complex and the target protein, a silver colloid was added to the resulting mixture, and the SERRS spectra were measured. With a decrease in the bovine serum albumin concentration, a decrease in the characteristic band associated with the chelate complex was observed. A sensitive SERRS detection of estrogen using the coupling reaction between phenolic estrogens and Pauly’s reagents was proposed [122]. The one-step and simple method included mixing the analyzed hormone with Pauly’s reagents (azo coupling) and adding the mixture to a solution of silver nanoparticles, followed by the registration of concentration-dependent SERRS spectra.

SE-SRS is a promising tool for studying the ultrafast dynamics of the molecules adsorbed on a substrate with a high temporal and spectral resolution. Obtaining effective plasmonic SERS substrates that are stable under the conditions of a femtosecond laser and provide a flexible choice of an adsorbate molecule is an urgent task for the development of the method. Negru et al. proposed a protocol for the preparation of oligomers of gold nanospheres using polyvinylpyrrolidone to stop the oligomerization of the monomer, and the effectiveness of the synthesized substrate was confirmed by the study of a number of molecules, including β-carotene, by the SE-SRS technique [123].

At the end of 2019, the world was faced with a pandemic caused by the SARS-CoV-2 virus. The need to quickly diagnose and control the spread of the virus gave impetus to the development of new methods for identifying the virus with high detection sensitivity. In this area, advances in the SERS technique have made it possible to adapt this method for direct virus testing. Acquisition of the reliable spectra of spike protein, nucleocapsid protein, and inactivated SARS-CoV-2 virion particles was achieved by the design of SERS-active substrates. Thus, the combination of Mxenes, possessing the advantages of metallic conductivity, hydrophilicity, tunable electronic structure, etc., with Nb and Ta cations, resulting in the formation of Nb_2_C and Ta_2_C MXene nanosheets, allowed for a sensitive detection of SARS-CoV-2 spike protein [124]. In another work, Sanchez et al. modified several layers of MoS_2_ with a mixture of Au–Cu nanostars and virion particles of the S and N proteins to determine the spike protein with nanomolar sensitivity [125].

Apart from the sensing application of direct SERS techniques, they have been successfully implemented for in vivo and ex vivo studies [31]. The study of biological tissues and fluids using SERS-based techniques is not an easy task, but several approaches have been proposed to solve it, such as the implementation of SERS substrates based on metal nanoparticles directly into the sample, applying a colloidal solution of the substrate to the sample, or freezing the sample with liquid nitrogen followed by grinding and mixing with the SERS substrate [126]. 

SERS is a promising tool for the nondestructive study of samples under physiological conditions. The potential of the SERS for ex vivo studies of cerebrospinal fluid has been shown by the example of identifying the meningitis pathogens [127]. The authors showed that by synthesizing a suitable SERS substrate—in this case, Au–Ag-coated polycarbonate membranes, which meets the requirements of a high enhancement factor and chemical stability—it is possible to achieve the label-free detection of the inflammatory marker neopterin, the level of which increases in the cerebrospinal fluid infected with *Neisseria meningitidis*, *Streptococcus pneumoniae*, and *Haemophilus influenzae*.

Achieving high sensitivity up to the detection of a single molecule using SE-SRS is challenging due to the potential for the damage of substrates by ultrafast pulses and a high pump–probe background. Compared to SERS, the SE-SRS technique, which provides an improved imaging speed, allows it to be used to identify chemical changes in living systems. Thus, Zong et al. achieved the single-molecule detection of adenine released from *S. aureus* cells as a result of starvation using aggregate gold nanoparticles dropped onto a glass surface [78]. The results obtained can be extended to the detection of biomarkers in tissues by using SERS nanotags containing bioreceptor molecules specific to the analyte.

The combination of antibody-modified SERS substrates with nonlinear optical microscopy (SE-CARS) is a promising approach for ex vivo and in vivo live cell imaging. Thus, Machtoub et al., using an ex vivo model of amyotrophic lateral sclerosis, investigated cellular metabolic states by the SE-CARS technique using iron oxide paramagnetic particles labeled with anti-CD4 antibodies [128,129]. It was shown that the high intensity of SE-CARS, caused by the binding of lipids to labeled antibodies, correlates with the identified areas of brain damage.

**Table 2:** SERS and SERS-combined techniques for direct label free biosensing.

Analyte	SERS Substrate	LOD	Laser Wavelength, Laser Power	Features	Year, Ref.
Pyruvate, adenosine triphosphate (ATP), and lactate	Fe_3_O_4_ microspheres-decorated silver nanoparticles (30 nm)	0.1, 0.01, and 1.0 pM for ATP, lactate, and pyruvate detection, respectively	632.8 nm, ≈7 mW	Simultaneous detection of multiple analytes, provided by a combination of a microfluidic SERS platform and magnetic separation that creates hot spots.	2019, [130]
Pyocyanin secreted by *Pseudomonas aeruginosa*	Microchannels made of poly (dimethylsiloxane) (PDMS) with integrated gold nanooctahedrons	10^−19^ M	785 nm laser, 1.74 mW	Detection of biomolecules without extraction from complex biological media using SERS-based microfluidic chip.	2020, [131]
Thiram and carbaryl detection	Poly(ethylene terephthalate) covered with indium tin oxide and silver layers	2.5 µg/mL for thiram and 0.012 µg/mL for carbaryl	785 nm, 5 mW	Processing the foil with the dielectric barrier discharge method to create a rough surface and subsequent modification with silver nanoparticles allows for the fabrication of a flexible SERS substrate for analysis.	2019, [132]
Dopamine	Two-dimensional WS_2_ grown on three-dimensional WO_3_ nanohelixes by sulfurization process	10 nM	Ar-ion 633 nm	Application of two-dimensional dichalcogenides of transition metals in the construction of a substrate for the detection of biomolecules.	2020, [133]
Manganese super oxide dismutase (MnSOD)	Glass substrate, modified with gold nanoantennas and thiolated DNA aptamer with specificity to MnSOD protein	Nanomolar range	660 and 785 nm, 1 mW	Combination of the high sensitivity provided by gold nanoantennas with the specificity provided by the modification of the substrate with an aptamer.	2015, [134]
Bilirubin	A hybrid of graphene oxide and gold nanostars applied to filter paper	0.436 μM	785 nm, 6.57 mW	Physical self-assembly between graphene oxide and gold nanostars to obtain SERS active substrate.	2019, [135]
Bacteria mixture (*Shigella flexneri*, *Escherichia coli* O157:H7, *Staphylococcus aureus*, and *Listeria monocytogenes*)	Silver nanoparticles	1.5 cfu/mL	He–Ne 632.8 nm, 14 mW	Homogeneous SERS detection of bacteria based on the binding of bacteria to the aptamer, followed by the in situ synthesis of silver nanoparticles on the bound aptamer.	2017, [136]
Carbapenem-sensitive *E. coli* (CSEC) and carbapenem-resistant *E. coli* (CREC)	Silver-coated gold nanorods (Au@Ag NRs)	Identification of CSEC and CREC	785 nm, 20% of the laser power	Homogeneous SERRS identification of bacteria, where the plasmon peak of the Au@Ag NRs nanostructure coincides with the laser wavelength, which provides the necessary sensitivity due to the resonance enhancement effect.	2018, [137]
Biomarkers of breast cancer in human tears	Gold-decorated, hexagonal-close-packed polystyrene nanosphere monolayer	Identification of breast cancer markers	785 nm, 10 mW	SERS biosensor for identification of breast cancer biomarkers, which are predictors of disease, based on the analysis of human tear spectra.	2020, [138]

#### 2.1.3. Raman Reporter Molecules

To achieve a high performance of indirect SERS, the choice of a reporter molecule with a large Raman scattering cross section is also important. The distance between the Raman reporter molecule and the SERS substrate and the SERS nanotag is an important parameter contributing to the enhancement factor because the distance between the reporter molecule and the SERS substrate/nanotag makes the main contribution to the electromagnetic enhancement and the adsorption on the surface to the chemical enhancement. Fluorophores and chromophores, such as rhodamine, methylene blue, malachite green isothiocyanate, and crystal violet, as well as thiol-containing aromatic compounds (5,5′-ditho-bis (2-nitrobenzoic acid, DTNB), 4-nitrothiophenol, 4-aminothiophenol, 4-mercaptobenzoic acid, etc.), meet these requirements [139,140]. Most often, preference is given to thiol-containing compounds, which is explained by their high binding ability to the nanostructure surface, ensuring the close position of the reporter molecule to the metal surface and, accordingly, a high SERS electromagnetic EF. Along with the widely used reporter molecules that form an Au–S bond when interacting with metals, a new class of compounds of aryl diazonium salts, forming metal–C interfacial bonds, has been proposed [141]. The special advantages of this class of compounds are the ability to form several layers of reporters around the metal core with different functions. Recently, Javaid et al. [142] synthesized a new class of boron complexes based on pyridine–pyrazole ligands and proposed their use as reporter molecules. Evaluation of the SERS effect of Raman active dyes on the surface of gold nanoparticles with a size of 40 nm showed the efficiency of boron complexes with the retention of sharp characteristic peaks at a low concentration of the reporter molecule, which is an advantage for their potential use in quantitative analysis.

#### 2.1.4. Protection Strategies for SERS Nanotags

To prevent the desorption of the Raman reporter molecule from the surface and the binding of other molecules to the SERS nanotag, metal nanoparticles modified with the reporter molecule are often coated with a nanoshell. The most common compounds for the formation of a protective shell are polyethylene glycol derivatives, silica, and bovine serum albumin [143,144,145]. Kim et al. used HS-PEG-COOH both to protect gold nanoparticles modified with Raman reporter molecules and to provide binding with antibodies through EDC/NHS coupling chemistry [146]. The formation of the Au–S bond between thiol-PEG and gold nanoparticles stabilized the structure of the SERS nanotag. The combination of magnetic beads as a capture substrate and two SERS nanotags containing Nile Blue A and malachite green isothiocyanate made it possible to detect botulinum toxins A and B. Silica encapsulation is one of the common protection strategies that, due to the ease of modification, biocompatibility, and stability, allows the obtained SERS nanotags to be used for analysis in biological matrices and in a multiplex analysis format [147]. Neng et al. synthesized silica-encapsulated SERS nanotags for the simultaneous detection of pathogenic antigens from West Nile virus, Rift Valley Fever virus, and *Yersinia pestis* using magnetic nanoparticle-based capture substrates [148]. 

To ensure the stability of the SERS nanotag to environmental interferents and significantly improve the signal intensity resulting from the formation of hot spots, researchers proposed a core-shell configuration of SERS nanotags, where the Raman reporter molecule is embedded in the gap between the metal core and the shell [145]. Recently, Duffield et al. have optimized the synthesis of silver-coated gold nanoparticles, between which DTNB is embedded, as well as bioconjugation conditions for the use of SERS nanotags in biomedical settings [149]. The authors showed that with an increase in the thickness of the silver shell, the SERS signal was enhanced. Compared to the widespread Ag@Au and Au@Ag, where the core is a spherical Au/Ag nanoparticle, the synthesis of nanorod-shaped metal nanoparticles as a core with a SPR closer to the near infrared region is of greater interest for biosensor development. Thus, Khlebtsov et al. [150] obtained Au@Ag nanorods, the so-called “anisotropic nanomatryoshkas,” and they demonstrated that by varying shell thickness, one can adjust the enhancement effectiveness. In addition, a study was carried out on the influence of the position of the Raman reporter molecule in the SERS nanotag (positioned between the core and the shell or adsorbed on the shell), and it was shown that the protection of the reporter molecule by the metal shell provides a strong and reproducible SERS signal.

#### 2.1.5. Capture Receptor Molecules for Indirect Approach

The specificity of the SERS nanotag toward the molecule of interest is provided by the conjugation of the nanotag to the bioreceptor molecule. To date, there is a range of bioreceptor molecules for designing the SERS nanotag to specifically target the molecule of interest, including antibodies and aptamers. The choice of antibodies is preferable because it allows high-affinity specific binding to the target analyte [151]. SERS-based immunoassay techniques are described to determine bacteria [152], viruses [153], immunoglobulins [154], cancer biomarkers [110,155,156], infection diseases [157], and so on. 

**Figure 5:**
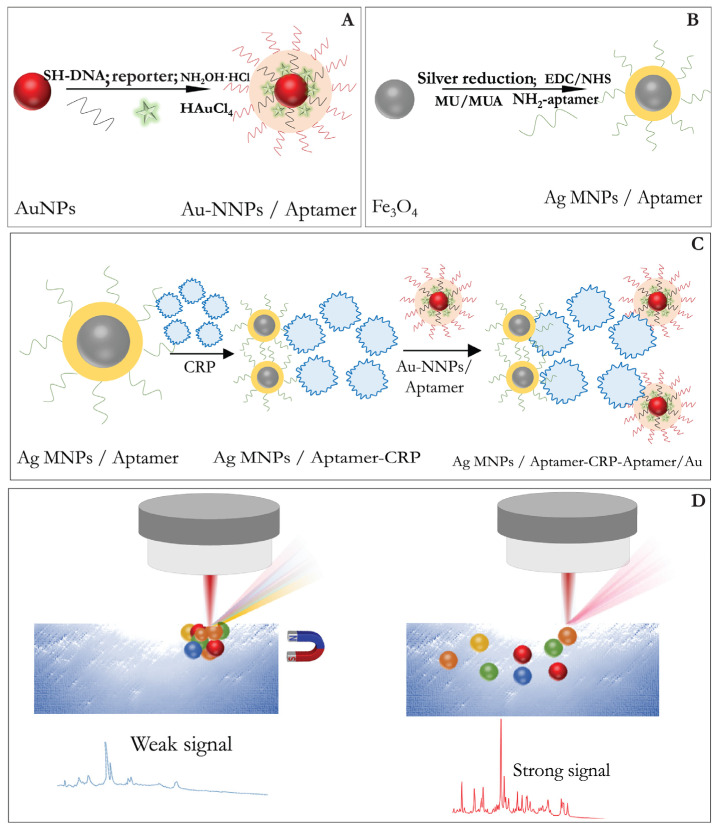
Schematic illustration of SERS aptasensor for CRP detection based on AgMNPs and Au-NNPs as SERS nanotag and magnetic capture substrate, respecitvely. The analysis included the following steps: (**A**) fabrication of SERS nanotag; (**B**) obtaining of AgMNPs modified with aptamer; (**C**) incubation of the CRP with Ag MNPs and SERS nanotag resulting in the formation of the sandwich complex AgMNPs–CRP–Au–NNPs; (**D**) magnetic separation of unbound SERS nanotag followed by SERS detection.

In the implementation of aptamer-based analysis, a sandwich scheme is used when both the SERS-active substrate and the SERS nanotag are functionalized with complementary chains of single-stranded DNA or RNA molecules to form a sandwich complex with the target molecule [158]. These assays have different sequence of stages. Figure 5, drawn in the review, integrates the assay stages for the case, where two kinds of nanoparticles are used as (i) a SERS substrate and a carrier for receptor moleclules, and (ii) a carrier for reporter and receptor molecules. The proposed aptasensor detects C-reactive protein. The primary NH_2_-functionalized aptamer, was immobilized on silver-coated magnetic nanoparticles (AgMNPs) and used as a capture substrate. Gold-coated magnetic nanoparticles (AuMNPs) modified with reporter molecules and secondary SH-functionalized aptamer, were applied as a detection probe. After the incubation of the CRP with the substrate, the complex AgNPs–CRP was magnetically separated. Thereafter, a detection probe was added to form a sandwich complex, “AgMNPs-CRP-AuMNPs,” the signal of which was recorded after the magnetic separation of the unbound AuMNP-based SERS nanotag. The described scheme of the aptasensor enabled the detection of CRP at a fM concentration range. The indirect sandwich SERS aptasensors were successfully developed for the detection of the insulin-like growth factor 2 receptor protein [159], 17β-estradiol [160], prostate cancer specific pca3 mimic DNA [161], C-reactive protein (CRP) [162], etc.

In recent years, there has been a growing interest in the use of antibody-mimic molecularly imprinted polymers (MIPs) that are prepared through copolymerization in the presence of a template [81]. MIPs are a cost-effective replacement for antibodies that are easily prepared and stable under harsh analytical conditions. Recently, the high sensitivity of the SERS sensors has been demonstrated, provided by the use of MIP-modified SERS substrates [87,88]. For example, Xing et al. first applied MIPs in dual MIP-based plasmonic sandwich assay for neuron-specific enolase detection in human serum [163]. A self-assembling layer of boronic acid-functionalized gold nanoparticles was formed on a glass substrate, and the glycated C-terminal epitope was immobilized through its affinity to boronate. Boronic acid-functionalized silver-coated silica nanoparticles were modified by reporter molecules and N-terminal glycated epitopes. Imprinting layers are formed directly on the surface of the SERS substrate and nanotag by the polycondensation of several silylating reagents containing functional groups capable of interacting with epitope sequences. After the glycated epitopes are washed with an acidic solution of acetonitrile, which destroys non-covalent and boronate bonds, the C-terminal epitope imprinted AuNP-modified glass slide is used as a substrate for capturing a biomarker from complex biological samples, and the SERS nanotag with imprinted N-terminal epitopes is used as a detection probe. Zhu et al. [164] proposed horseradish peroxidase-induced in situ polymerization of MIP-SERS substrate using Au/poly (dimethylsiloxane)/anodized aluminum oxide SERS substrate, patulin as a template, 4-vinyl pyridine as a functional monomer, and 1,4-diacryloylpiperazine as a cross-linker. The effectivity of synthesized MIP-SERS substrate was confirmed by selective and sensitive detection of patulin in real samples. 

The choice of bioconjugation method depends on the terminal groups of the ligand on the surface of the SERS substrate. Bioconjugation of substrates containing carboxy or amino groups on the surface is carried out by covalent immobilization with the formation of an amide bond. For example, for silica-encapsulated SERS nanotag, thiol or amino-containing silanes with a bifunctional linker are used to bind to the receptor molecule [165]. In the absence of a stabilizing layer, both simple adsorption of bioreceptor molecules on the surface of the SERS substrate and covalent immobilization can be realized. A review of the applied methods of the conjugation of bioreceptor molecules with SERS substrates is given in the work of Langer et al. [86].

## 3. Recent Advances of Raman Spectroscopy in Biosensing

To date, the SERS technique is a promising analytical tool that provides a nondestructive and minimally invasive method for in vitro and in vivo analysis, requiring minimal sample preparation. The first and most widespread application of the SERS method for bioanalytical purposes is the immunoassay of various protein markers in biofluids [81,140]. Table 3 summarizes the recent research on the application of indirect SERS-based techniques in homogeneous and heterogeneous formats of biosensors. The homogeneous format of SERS detection implies a mandatory stage of separation of immune complexes and unreacted components, including the unbound SERS nanotag. For this purpose, magnetic nanoparticles are usually used [166,167]. Recently, a new scheme of SERS-based biosensor was proposed, in which the SERS active product is synthesized as a result of the formation of an immunocomplex [166]. The use of nanoparticles simultaneously with enzymes allowed for combining the functions of the carrier of active components of immunoassay (antibodies, antigens) and the enzymatic transformation of substrates [168]. The substrate of alkaline phosphatase, the so-called 5-bromo-4-chloro-3-indolyl phosphate (BCIP), in the immunoassay is hydrolyzed to the inorganic phosphate and sole active compound 5-bromo-4-chloro-3-indole (BCI) [169,170]. The resulting product of the reaction has characteristic bands detected during the SERS immunoassay.

Pham et al. proposed an enzyme-amplified SERS immunoassay for the detection of IgG and prostate-specific antigen. The principle of the method is based on the enzyme-catalyzed reduction of silver in the form of a shell on Au NP-assembled silica NPs modified by a Raman molecule. In the presence of the target analyte, the immune complex labeled with the enzyme alkaline phosphatase triggered the reaction of 2-phospho-l-ascorbic acid into ascorbic acid (AA). In turn, ascorbic acid reduces Ag^+^ to Ag, forming hot spots that enhance the signal from the SERS of the sample solution. Another example of the formation of the SERS active product is described as the highly sensitive detection of allergens by the SERS-based ratiometric enzyme-linked immunosorbent assay [171]. In the current study, antibody-modified AuNPs doped with a covalent organic framework were applied as the SERS nanotag. This SERS nanotag showed the properties of nanozymes and catalyzed the reduction of a 4-nitrothiophenol substrate with further reduction in the presence of sodium borohydride to 4-aminothiophenol. The latter formed the Au–S bond with gold nanostars, resulting in the formation of Raman hotspots. The concentration of the analyte under study was determined from the ratio of the signal intensities of the characteristic bands of 4-aminothiophenol and 4-nitrothiophenol, respectively. Compared to fluorescence and luminescence, the Raman peaks are narrower, which contributes to the implementation of the multiplex format of SERS analysis [140]. In particular, a multiplex SERS-based immunoassay for pathogen [148], cancer marker [172,173], and toxin [174] detection has been implemented recently. New platforms and advances are discussed below that expand the potential application of SERS biosensors for rapid and point-of-care analysis.

**Table 3:** Indirect biosensing using SERS nanotags.

Analyte	SERS Substrate/Receptor Molecule	Assay	SERS Nanotag	LOD	Sample	Features	Year, Ref.
Gp51 antigen of bovine leukemia virus	Magnetic gold nanoparticles (AuNPs)/the native (anti-gp51) and fragmented anti-gp51 antibody (Ab)	Homogenous SERS-based sandwich immunoassay	Gold nanorods modified with 5-thio-nitrobenzoic acid (DTNB) and specific anti-gp51 Ab	0.95 μg/mL	Milk	Oriented and random Ab immobilization, application of two kinds of nanoparticles	2013, [175]
*Escherichia coli* (*E. coli*)	Gold-coated magnetic spherical nanoparticles/polyclonal antibody (pAb)	Homogenous SERS-based sandwich immunoassay	Rod shaped AuNPs modified with DTNB, avidin, and biotin-labeled Ab	8 cfu/mL	Real water samples	Two kinds of AuNPs	2011, [153]
*E. coli* and *Staphylococcus aureus* (*S. aureus*)	Magnetic beads (400 nm)/anti-*E. coli*2, anti-*S. aureus*2 monoclonal antibody (mAb)	Homogenous SERS-based sandwich immunoassay	Poly-l-lysine-coated triple-bond-coded AuNPs modified with 4-cyanobenzenethiol (MBN)	10 and 25 cfu/mL	Bottled water and milk	Simultaneous detection with “hot spot” effect resulting in a significant enhancement of the Raman signal at 2105 and 2227 cm^−1^	2020, [152]
Human immunoglobulin (hIgG)	100 nm thick gold film evaporated on microscope slide or silicon wafer/goat anti-human IgG Ab	SERS immunoassay of human immunoglobulin	60 nm gold nanoparticles modified with 4-nitrobenzenethiol (4-NBT) and anti-human IgG Ab	3 pM on silicon and 28 pM on gold	Standard solution	Comparison of Si wafer and tradition gold surface	2020, [154]
Human IgG, prostate-specific antigen (PSA)	2D arrays of Au (42 nm-core)@Ag (4.5 nm-shell) NPs on ITO substrate/polyclonal anti H-IgG, PSA mAb	Heterogenous SERS-based sandwich immunoassay	SH-PEG-COOH-coated AuNPs modified with 4-mercaptobenzoic acid (MBA) and anti H-IgG or PSA mAb	0.3 pg/mL (10 fM) for PSA and 0.05 pg/mL (0.3 fM) for H-IgG	Standard solution	Comparison of the size of AuNPs in SERS nanotag (26, 53, 110 nm)	2017, [155]
*Escherichia coli* (*E. coli*)	Spherical gold coated magnetic nanoparticles/pAb	Homogenous SERS-based sandwich immunoassay	Gold nanorods labeled with alkaline phosphatase (ALP) enzyme and also modified with 5-bromo-4-chloro-3-indolyl phosphate (BCIP) and *E. coli* Ab	10 cfu mL^−1^	Standard solution	ALP activity; BCIP was hydrolyzed to SERS-active product; 5-bromo-4-chloro-3-indole (BCI)	2018, [166]
IgM and IgG to SARS-CoV-2	No SERS substrate/mouse anti-human IgM and IgG capture Abs	SERS-based LFIA	Gap-enhanced Raman nanotags (GERTs) with 4-nitrobenzenethiol (4-NBT) between core and shell, modified with COVID-19 recombinant antigens (CN97)	1 ng/mL (IgM), 0.1 ng/mL (IgG)	Standard solution	Simultaneous determination of IgM and IgG	2021, [176]
IgM and IgG to SARS-CoV-2	No SERS substrate/anti-human IgM and anti-human IgG Abs	SERS-based LFIA	Ag shell on SiO_2_ core (SiO_2_@Ag) 5,5-dithiobis-(2-nitrobenzoic acid) modified with dual layers of DTNB and SARS-CoV-2 spike (S) protein	1.28 × 10^7^-fold dilution by the IUPAC standard method, which is 800 times lower than that of the visualization results	Clinical serum samples (*n* = 68)	Simultaneous determination of IgM and IgG	2021, [177]
Ferritin (FER)	Hydrophilic AgNPs onto the specific area of the hydrophobic polydimethylsiloxane (PDMS)–hydrophilic/hydrophobic Ag/PDMS/anti-FER Ab	SERS-based LFIA	Raspberry-like AuNPs modified with 4-MBA and anti-FER Ab	0.41 pg/mL	Standard solution	Combination of SERS substrate and SERS nanotag in LFIA format	2020, [178]
Carcinoembryonic antigen (CEA)	Hydrophilic AgNPs with polymethylmethacrylate (PMMA)/anti-CEA Ab	SERS-based LFIA	Flower-shaped Ag nanoplates modified with crystal violet and anti-CEA Ab	4.92 pg/mL	Standard solution	Combination of SERS substrate and SERS nanotag in LFIA format	2021, [156]
α-Fetoprotein (AFP)	Few layers of MoS_2_ nanosheets exfoliated by NaK alloys/capture mAb	SERS-based sandwich immunoassay	Au@AgNCs and R6G–mAb complex	0.03 pg/mL	Human blood serum samples	The sandwich immunocomplex “capture probe/target/SERS tag” was deposited on a silicon wafer and decorated with silver-coated gold nanocubes to increase the density of “hot spots” on the surface of the immunosensor	2021, [110]
Human immunoglobulin (hIgG)	AuNP array (AuA)-coated solid substrate/rabbit anti-human IgG Ab	SERS-based sandwich immunoassay	AuNPs modified with 4-aminothiophenol (4-ATP) and rabbit anti-human IgG Ab	0.1 μg mL^−1^	Human serum samples	The combination of a SERS substrate based on AuNP array with SERS nanotag resulted in sensitive detection	2021, [109]
Pancreatic cancer marker MUC4	Immobilization of gold nanoflowers onto thiol-functionalized silicon wafer/Anti-MUC4 Ab	SERS-based sandwich immunoassay	Gold nanoflowers modified with 4-mercaptobenzoic acid and anti-MUC4 Ab	0.1 ng mL^−1^	Standard solution	Raman mapping was applied for a large substrate area to decrease a “spot-to-spot” variation of SERS signal	2020, [179]
IgG/PSA	No SERS substrate/anti-rabbit IgG/anti-PSA Ab	Homogeneous enzyme-amplified SERS immunoassay	AuNP-assembled silica NPs (SiO_2_@Au-RLC@Ag) with Ag shell modified with 4-aminothiophenol (4-ATP) Polyclonal alkaline phosphatase (AP)-conjugated goat anti-rabbit IgG or AP-streptavidin-biotin-conjugated anti-PSA Ab were used as a tracer Ab to produce ascorbic acid for reduction of Ag^+^ to Ag	0.09 ng/mL for IgG and 0.006 ng/mL for PSA	Human serum samples	Enzyme-induced Ag growth on the surface of SERS nanotag to produce the amplification of the SERS signal	2020, [180]
Carcinoembryonic antigen (CEA)	Silver shell magnetic nanoparticles Fe_3_O_4_@Ag MNPs/anti-CEA monoclonal antibody	SERRS-based sandwich immunoassay	Silver-coated gold nanorods (Au@AgNRs) modified with diethylthiatricarbocyanineiodide (DTTC), coated with mPEG-SH and conjugated with anti-CEA antibodies	4.75 fg/mL	Human serum samples	Au@AgNRs were in resonance with the resonant Raman dye DTTC at 785 nm excitation laser	2016, [181]
Mannose-capped lipoarabinomannan (ManLAM)	Resonance Raman-enhanced adlayer of cyanine 5 on a smooth gold surface/polyclonal rabbit antibody for *Mycobacterium tuberculosis*	SERRS-based sandwich immunoassay	AuNPs modified with 5,5′-dithiobis (succinimidyl-2-nitrobenzoate; DSNB) and MAb to ManLAM	1.1 ng/mL	Human serum samples	Cy5 modified gold substrates were characterized; the SERRS performance was compared with SERS and revealed a ≈9.3 gain in sensitivity of immunoassay	2019, [182]

### 3.1. Microfluidic SERS-Based Biosensors

One of the directions for improving SERS technique is the combination of detecting devices with microfluidic approaches [183]. Microfluidics allow the researcher to increase analysis productivity by separating reagent streams and simultaneously detecting multiple analytes on a single chip. In addition, the use of microchannels minimizes sample volumes, and the complex configuration of interlaced channels allows for controlled flows, the uniform mixing of reagents, and improved reproducibility of results [184,185]. On the one hand, volume minimization is an obvious improvement in analytical techniques. On the other hand, it imposes more stringent requirements on the sensitivity of tag detection. Therefore, the combination of microfluidics with such a highly sensitive method as SERS seems to be the most logical option. In addition, microchannel detection allows for a high surface-to-volume ratio to be achieved, which is critical for efficient SERS measurements, whereas in-stream analysis reduces sample heating, which is one of the significant limitations of Raman techniques [183,186]. This harmonious combination of the two technologies has given rise to growing interest in the development of microfluidic analytical devices with SERS detection.

**Figure 6:**
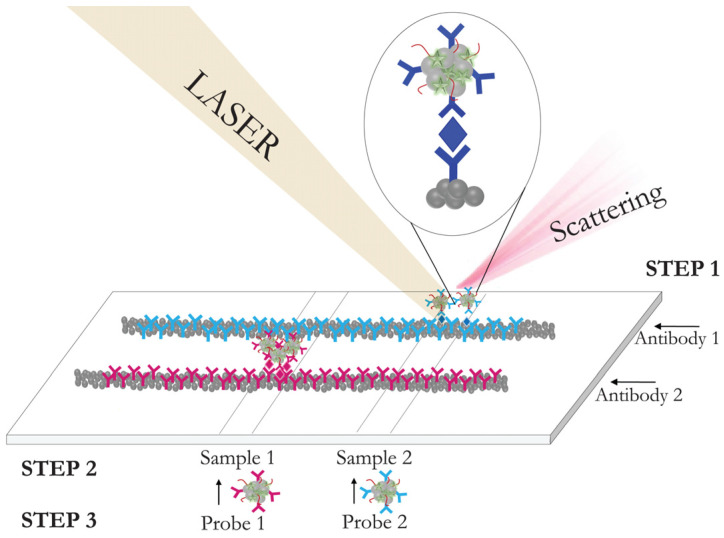
SERS microfluidic chip with AgNPs in microchannels, functionalized with capture antibodies. After injection of analyzed samples and SERS nanotags, the sandwich complexes are formed in microchannels, and SERS signals indicate presence of corresponding analytes.

To date, such analytical devices are successfully adapted for the detection of a wide range of analytes, including medicines and drugs [185,187], pesticides [188,189], hormones [190], antibiotics [191], disease markers [184,192,193,194], nucleic acids [195], whole cells [196], and others [186]. Figure 6, drawn in the review, represents the basic principle of measurements that is used in the listed above developments and its application to multi-analysis cases. When individual compounds are detected, the detection limit reaches the fg/mL range, and when detecting cells—single cells in milliliters [196]. The capabilities of separating streams and creating capillary matrices in microfluidic systems are perfectly combined with the capabilities of the simultaneous detection of multiple tags (thanks to the characteristic “fingerprints” of SERS nanotags). This opens up wide possibilities for creating systems for the multiplex detection of various analytes. Chen et al. developed a microfluidic matrix for the simultaneous detection of four markers of inflammation: C reactive protein, interleukin-6, serum amyloid A, and procalcitonin, using labels with SERS-encoded core-surface structures [192]. Multiplex detection can be useful for other tasks as well. For example, Wang et al. simultaneously used three different SERS-labeled molecular probes targeting different epitopes of the same pathogen to ensure the detection of pathogenic targets at the level of individual cells with subspecies specificity [196].

### 3.2. Integration of SERS with Different Methods

Recently, there has been a tendency in which (i) the conditions for the analysis become more complicated, and (ii) it has become possible to use two methods for determining the analyte content at once. The proposed strategy is a dual detection approach that combines several methods for determining the content of the target analyte or cells, which enables the comparison of approaches under the same conditions in terms of sensitivity or detection limit. Recently, SERS-combined techniques have been summarized in a review by Zhang et al. [197]. Thus, SERS was experimentally integrated with electrochemical techniques [198], fluorescence detection [199], and the polymerase chain reaction (PCR) method [200]. Thus, in the work of Wang et al. [199], nanostars modified with antibodies and aptamers were used to identify circulating tumor cells. Gold nanoflowers were arranged in an orderly manner on the Au-ITO electrode as a substrate and then modified with an aptamer interacting with circulating tumor cells. Nanostars treated with polyethylene glycol followed by the immobilization of two hairpin trigger DNA molecules with fluorescent and reporter tags, as well as antibodies against the determined cells, were used as the SERS nanotag. After the complex was formed on the substrate, it was possible to estimate its amount by the fluorescence intensity or by the Raman shift. When comparing two analytical methods (fluorescence and SERS), the authors showed a twofold reduced limit achieved by SERS detection. Castaño-Guerrero et al. [198] combined the possibility of the simultaneous electrochemical and SERS detection of specific antigen–antibody interactions in the determination of cancer embryonic antigen. Here, the authors used a specific antigen–antibody interaction in a sandwich immunoassay format. The first capturing antibodies were covalently immobilized onto gold screen-printed electrodes modified with cysteamine to form accessible, functional groups for crosslinking with antibodies. The second antibodies were immobilized on gold nanostars together with a reporter molecule (4-ATP). Electrochemical impedance spectroscopy was performed by the authors prior to the addition of the Raman probe to assess the capabilities of electrochemical detection in this format. Thus, the analysis itself was carried out in two stages, and both methods of detection were separated in time. The authors point to a 10-fold gain in sensitivity when using SERS; however, when switching from buffer to serum containing the cancer embryonic antigen, the detection limit dropped by 10 times, which was probably due to the impossibility of avoiding the matrix effect of the blood serum sample.

The dual mode of determining three types of bacteria (*Escherichia coli*, *Salmonella*, *Staphylococcus aureus*) in contaminated milk, with the possibility of qualitative and quantitative determination, was demonstrated by Xu et al. [201]. In this work, two types of nanoparticles were applied: functionalized upconverting nanoparticles as a fluorescent probe and bimetallic (Au@Ag) magnetic nanoparticles modified with 4-mercaptophenylboronic acid (4-MPBA) as a SERS tag. Interestingly, for the interaction, the authors chose a phenylboronic acid derivative, which can be used for binding with the carbohydrate moiety of the wall bacteria. Here, 4-MPBA, as both a SERS reporter and bacteria capturer, was applied for the development, and there was no need to use additional reporters to form a Raman probe. Despite the fact that three different species of bacteria bind the same to the phenylboronic acid residue, the SERS reporter spectra differ for these species. The authors called this the fingerprints of bacteria, that is, the individual differences of bacteria made their own changes in the spectral characteristics of the bacteria used, which made it possible to use this parameter to identify the bacteria in the mixture. Thus, the above study does not so much show the advantage of fluorescence or SERS, but rather shows how this method and the corresponding reporter can be used, as well as how the signal changes depending on whether the bacterium is alive or killed.

Lee et al. [200] proposed the integration of PCR with paper-based SERS for the detection of the bacterial DNA of *Mycoplasma pneumoniae* as a model. At the first stage, it was necessary to accumulate bacterial DNA by PCR. Silver nanowires were applied to the surface of the paper-based device. In the presence of the target DNA, after several cycles of amplification (0–30), the applied EvaGreen dye was reversibly incorporated into the DNA structure. In this case, the observed SERS signal was low. If the desired DNA was not formed in the process, then the dye would adhere to the silver nanowires on the surface of the paper to form so-called hot spots, and the signal was amplified accordingly. In this case, the SERS signal was read from the surface of the paper. The difference between a positive and a negative result was observed when comparing the intensities at the test and control points.

### 3.3. SERS-Based Lateral Flow Immunoassay

The development of rapid point-of-care systems for the determination of various compounds is a priority direction in life science research. Among the existing approaches, lateral flow immunoassay (LFIA) tests based on antigen–antibody interactions in a flow of reagents on a membrane carrier with the formation of visually detectable nanoparticle-labeled immune complexes are in demand. The indisputable advantages of LFIA are its low cost and ease of testing. However, insufficient sensitivity and insufficiently high-quality (“yes/no”) results reduce the effectiveness of test systems. In this regard, the integration of LFIA with SERS detection is of great interest because capture antibodies immobilized in the test zone concentrate the SERS nanotag, and the lateral flow format of assay allows the study to be carried out without additional washing and separation steps [202]. The recent applications of SERS-based LFIA for target marker detection are shown in Table 3. 

**Figure 7:**
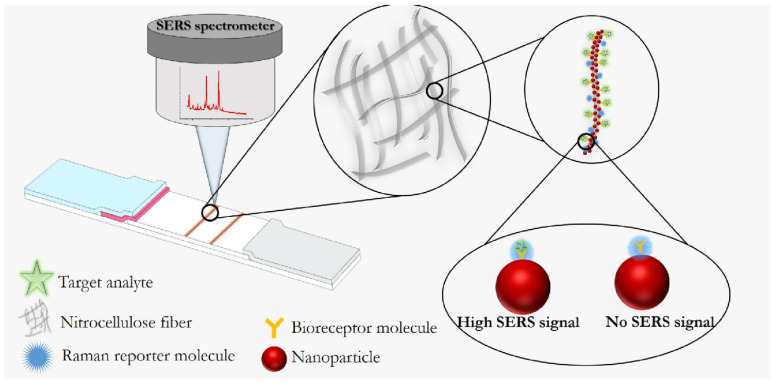
Principle of SERS-based LFIA.

The schematic illustration of SERS-based LFIA is given in Figure 7. For the assembly of SERS-based LFIA, antibodies and aptamers (oligonucleotide sequences) are used as receptor molecules to interact with the target compound. The choice of antibodies is preferable because it provides high-affinity specific binding to the target analyte. Nanoparticles of noble metals (most often gold nanoparticles), which are used in traditional LFIA as a detectable label and in SERS immunoassay as part of the nanotag, combine these two analytical methods. This is because the modification of nanoparticles with a reporter and receptor molecules leads to the formation of a SERS nanotag. The result of the interaction of the selected receptor molecules with the analyte will be a change (increase or decrease) in the amount of SERS nanotags bound in the test zone. The spectra of reporter molecules from SERS nanotags make it possible to identify the content of analytes during the formation of an immune complex. Previously, the strategies to design high-performance SERS-based LFIA systems and prospects for their application were summarized in the review by Khlebtsov et al. [203].

In the design of the SERS-based LFIA, an important parameter is the shape of the metal nanoparticles in the SERS nanotag [204]. Sánchez-Purrà et al. [204] provided the comparison of gold nanoparticles of different shapes for the detection of viruses in LFIA. According to the study, nanoflowers, nanostars, and other particles with sharp spikes and corners potentially have advantages for use in SERS-based LFIA because they can have higher signal amplification due to the redistribution of electric fields at the ends. In this regard, it is preferable to use the formed nanoparticles in systems with low sensitivity to improve the performance of the analysis. For example, Lin et al. compared the performance of SERS-based LFIA for the determination of biphenol A using the SERS nanotags based on star-shaped and spherical gold nanoparticles of the same size (40 nm) [205]. The authors showed that the use of SERS nanotags, including gold nanostars modified with 4-ATP and anti-BPA antibodies, can increase the visual and quantitative detection limit of bisphenol A by 20 and 205 times, respectively, compared to gold nanospheres.

A similar efficiency of star-shaped gold nanoparticles was shown for the determination of the nucleoprotein of the influenza virus by the SERS-LFIA method [206]. In this work, the star-shaped nanoparticles with a size of about 100 nm, modified with 4-ATP and monoclonal antibodies specific to nucleoproteins, were used as a SERS nanotag. The detection limit for the influenza virus nucleoprotein was 37 and 300 times better than the characteristics of fluorescent and standard LFIA, correspondingly. The size of nanoparticles also plays an important role in the development of SERS-based LFIA. Chen et al. [176] demonstrated that it is preferable to use particles larger than 40 nm due to the stronger effect of surface electromagnetic amplification and as a result of the higher SERS signal. However, in the synthesis and selection of the SERS nanotag, the most important factors are the stability of the nanoparticles, the quality of the coating, the size of the gap, and the number of reporter molecules per particle, which can be detected using Raman spectrometers. 

Thus, Khlebtsov et al. synthesized gold nanorods covered with a gold shell with a gap between the core and shell of 1 nm and the included reporter molecule 1,4-nitrobenzenthiole (NBT), which, after conjugation with specific antibodies, were used as a SERS nanotag in the SERS-LFIA of troponin I [207]. The authors compared the sensitivity of the developed SERS-LFIA and the standard LFIA assembled with the same immunoreagents and showed a 30-fold gain in sensitivity when using the SERS nanotag in conjunction with the SERS signal registration. The efficiency of a 1 nm gap between the metal core and the shell was confirmed in another work [176], where the so-called gold “nanomatreshki” were obtained, consisting of a spherical gold core and a shell with a 4-NBT reporter molecule inserted between the layers. Gap-enhanced nanoparticles were functionalized with a recombinant antigen and used to develop SERS-LFIA for the simultaneous detection of anti-SARS-CoV-2 IgM and IgG antibodies. The sensitivity for IgM and IgG antibodies detection was two orders of magnitude higher than the characteristics of commercial LFIA tests for antibodies to SARS-CoV-2.

Tang and coauthors [156] proposed a new approach for improving the sensitivity of SERS-LFIA that consists in integrating a sandwich scheme of SERS immunoassay with LFIA. Thus, hydrophobic and hydrophilic regions were formed on the analytical membrane in the test and control zones, consisting of a hydrophobic polymethyl methacrylate layer with a sprayed hydrophilic silver layer on which antibodies to carcinoembryonic antigen (anti-CEA) were immobilized. The flower-shaped silver nanoplates modified with a crystal violet reporter molecule and anti-CEA antibodies were used as a SERS nanotag. Ultrasensitive analysis of carcinoembryonic antigen was achieved using the developed SERS-LFIA.

## 4. Toward Portable Raman Spectrometers

The widespread use of Raman spectroscopy is limited by the need for sophisticated and expensive equipment. Over the past decade, portable and relatively inexpensive Raman spectrometers have been manufactured with a view to making rapid and nondestructive on-site detection available. Table 4 summarizes the development of portable Raman analyzers and their applications for the identification of various objects. Portable spectrometers are equipped with a fixed optical head and a laser with adjustable power. The choice of a suitable laser avoiding fluorescent interference depends on the molecule to be identified. A number of studies have proven the effectiveness of handheld spectrometers for the detection of drugs [208,209,210] and different toxins [211]. Going forward, the miniaturization of equipment has also affected nonlinear optical microscopy techniques, CARS microscopy, and SRS microscopy. The practical implementation of CARS in clinical practice in the form of an endoscope or a miniaturized portable microscope is of great interest, but it is associated with a number of technical difficulties, summarized in the review by Tu and Boppart [212]. Overcoming technical complexities is possible by choosing between Raman spectroscopy over a large spectral range or imaging using single-frequency Raman lasers. Thus, several attempts have been made to design portable CARS imaging systems, including miniaturized CARS microscopes with a single femtosecond laser and custom optics [213] and all-fiber coherent Raman systems [214]. A handheld SRS microscope is also an essential tool for in situ chemical and biological imaging. Liao et al. [215] were the first to develop a miniature SRS microscope based on a time-separated pump and Stokes pulses that ensure there is no nonlinear background. The applicability of the developed SRS microscope was demonstrated by the chemical imaging of pesticide residues in spinach leaves and cancerous tissues and the study of several other objects.

The integration of LFIA test strips with portable SERS readout devices is a promising direction for point-of-care testing because it will expand the scope of rapid tests, enable multiplex assays, and improve their effectiveness by moving from qualitative to quantitative analysis. Thus, Tran et al. [216] constructed portable SERS-based LFIA for human chorionic gonadotropin. The design of the device included a fiber optical probe with a linear focus and a miniature 785 nm laser diode. The test zone of the LFIA strip was scanned using a motorized stage; the acquisition time for one strip was 5 s. The detection limit for hCG, achieved using a portable Raman spectrometer, was 15 times lower than that of commercial LFIA tests. Recently, Xiao et al. [217] reported a portable SERS-based LFIA for the simultaneous detection of alpha-fetoprotein (AFP), carcinoembryonic antigen (CEA), and prostate-specific antigen (PSA). The authors designed a multichannel LFIA reaction column with a 785 nm miniature diode laser and a stepper motor that moves the test strips inserted into the column in the desired direction. The Raman probe allows the automatic and point-by-point registration of Raman signals at several points of the test zone, whereas the acquisition time is 1 s for one test zone. The obtained values of the intensities of the Raman signal are averaged over all points using the developed software. Compared to ELISA, the proposed portable SERS-LFIA allows for more accurate and quick determination of biomarkers. However, with the transition to portable Raman spectrometers, a decrease in sensitivity is often noted in comparison with laboratory massive devices. Therefore, the design of portable handheld SERS-based LFIA tests combining the high sensitivity of the SERS technique and the rapidity of the LFIA remains an urgent direction of development.

## 5. Conclusions and Future Prospects

This review summarizes and presents the achievements of Raman spectroscopy methods and derivatives, CARS, SRS, RRS, and SERS, as well as their combination and association with bioanalytical and microscopic methods. Fundamental aspects of these methods are briefly summarized. The phenomenon of Raman scattering and approaches to its registration underlie spectroscopic research in various fields of life sciences, including biology, biochemistry, biotechnology, and medicine. Raman spectroscopy techniques are used for the noninvasive, label-free identification of molecules and the determination of their chemical composition and spatial orientation. In addition, they find their application for the hyperspectral imaging of the localization and number of molecules in biological objects and environments. CARS and SRS make it possible to increase the signal intensity at certain wavelengths that are in resonance with the natural vibrations of the molecule, which opens up additional opportunities for the highly selective spectral visualization of the objects under study. The limitations of nonlinear Raman spectroscopy and approaches to their resolution are briefly described. In this review, special attention is paid to the combination of SERS upon contact or close proximity of the target molecule with a metal substrate, with techniques of nonlinear Raman spectroscopy and RRS, and with immunochemical methods that ensure the detection of compounds with high sensitivity and specificity. This review summarizes the existing approaches for the formation of SERS substrates and nanotags, as well as schemes for the SERS-based analysis of molecules. The potential of using SERS-based techniques as a method for detecting immunochemical interactions is obvious and is supported by a number of successful developments discussed in this review. 

However, the use of SERS-based techniques in the detection and imaging of various molecules may be limited by several factors. First, the main problem is in measuring Raman spectra and applying this method for the quantitative analysis of a substance is associated with the inhomogeneous distribution of “hot spots” on the surface of the substrate, which contributes to significant changes in the reproducibility of the SERS signal. This problem of the preparation of SERS-active substrates is described by many authors who offer their solutions [218,219,220]; however, it has not yet been possible to develop a fundamentally new scheme for the fabrication of nanocomposite materials with the ideally uniform distribution of hot spots. Moreover, much attention needs to be paid to the study of a wider range of molecules of different origins, which find their application in life science [221]. The compilation of a public database of Raman spectra based on research data will provide the fast and reliable identification of unknown analytes by comparing the obtained data with the reference data. Along with this, in most works, there is no validation of the developed SERS-based biosensors. However, at present, isolated cases of the use of such techniques in clinical practice are already known, which indicates future high growth in the development of not only fundamental research but also technological applications in the medical and biological fields. Moreover, the ongoing development of standardized procedures for obtaining SERS substrates and nanotags that provide a high and reproducible signal, as well as miniaturization of spectrometers and the transition to portable devices for biosensing and imaging, are a huge step toward successfully removing the limitations of the Raman techniques.

## Figures and Tables

**Table 4:** Raman detection using portable devices.

Analyte	Substrate	LOD	Laser Wavelength, Laser Power	Features	Year, Ref.
Solid-dosage form medicines (62 formulations)	Direct measurement without SERS substrate	ND	785 nm, 270 mW	Positive identification of some generics and one placebo in comparison with instrumental method	2017, [208]
Cocaine mixtures (90 cocaine samples)	-	between 10 wt% and 40 wt%	785-nm, ≈250-mW	Direct identification in the mixtures with different content of cocaine, spectral identification by handled Raman spectrometer (comparison of results with GC–MS)	2021, [209]
In vivo imaging of the rat spinal cord, 20 µm and 4.5 µm polystyrene beads	Cover slip	-	≈800 nm, 300 mW	CARS microscope application for different assays including bioimaging	2010, [213]
Dermal structures in human and animal skin	Microscope cover slide without cover slip	-	tunable from 780 to 980 nm, 400 mW	Human skin tissues and mouse ear tissues were analyzed with lipid contrast by CARS microscopy	2019, [214]
Sample of mixed dried microspheres of PS and PMMA	Piece of paper	-	887 nm, 40 mW	Spectroscopic SRS microscopy, real-time hyperspectral SRS imaging	2018, [215]
Pesticide residue (thiabendazol) in spinach leaves	In situ label-free imaging	-	1040 nm, a tunable 80 MHz pulsed laser	SRS microscope in which a fiber delivered two laser pulses for imaging	2018, [215]
Human chorionic gonadotropin (hCG)	Au/Au core/satellite nanoparticles, Raman reporter molecule thio-2-napthol and the linker molecule (11-mercaptoundecyl)-*N*,*N*,*N*-trimethylammonium bromide	1.6 mIU mL^−1^	785 nm diode laser, up to 450 mW	Anti-hCG detection antibody was conjugated to the SERS nanotags	2018, [216]
Cancer markers (AFP, CEA, and PSA)	Gold nanorod nanotags functionalized with the Raman reporter molecule DTNB	0.01 ng/mL	785 nm diode laser, laser power range: 0–500 mW	SERS-based lateral flow immunoassay (LFIA) reader integrated with a multichannel LFIA reaction column, detection in human serum samples	2020, [217]
